# Immunity in the Cervix: Interphase between Immune and Cervical Epithelial Cells

**DOI:** 10.1155/2019/7693183

**Published:** 2019-04-17

**Authors:** Jorgelina Barrios De Tomasi, Michael Makokha Opata, Chishimba Nathan Mowa

**Affiliations:** ^1^Department of Biology, Appalachian State University, Boone 28608, USA; ^2^Departamento de Ciencias de la Medicina, Division de Ciencias de la Salud, Chetumal, Quintana Roo, Mexico; ^3^Rusangu University, Monze, Zambia

## Abstract

The cervix is divided into two morphologically and immunologically distinct regions, namely, (1) the microbe-laden ectocervix, which is proximal to the vagina, and (2) the “sterile” endocervix, which is distal to the uterus. The two cervical regions are bordered by the cervical transformation zone (CTZ), an area of changing cells, and are predominantly composed of cervical epithelial cells. Epithelial cells are known to play a crucial role in the initiation, maintenance, and regulation of innate and adaptive response in collaboration with immune cells in several tissue types, including the cervix, and their dysfunction can lead to a spectrum of clinical syndromes. For instance, epithelial cells block progression and neutralize or kill microorganisms through multiple ways. These (ways) include mounting physical (intercellular junctions, secretion of mucus) and immune barriers (pathogen-recognition receptor-mediated pathways), which collectively and ultimately lead to the release of specific chemokines and or cytokines. The cytokines subsequently recruit subsets of immune cells appropriate to a particular immune context and response, such as dendritic cells (DCs), T, B, and natural killer (NK) cells. The immune response, as most biological processes in the female reproductive tract (FRT), is mainly regulated by estrogen and progesterone and their (immune cells) responses vary during different physiological phases of reproduction, such as menstrual cycle, pregnancy, and post menopause. The purpose of the present review is to compare the immunological profile of the mucosae and immune cells in the ecto- and endocervix and their interphase during the different phases of female reproduction.

## 1. Introduction

**Figure 1:**
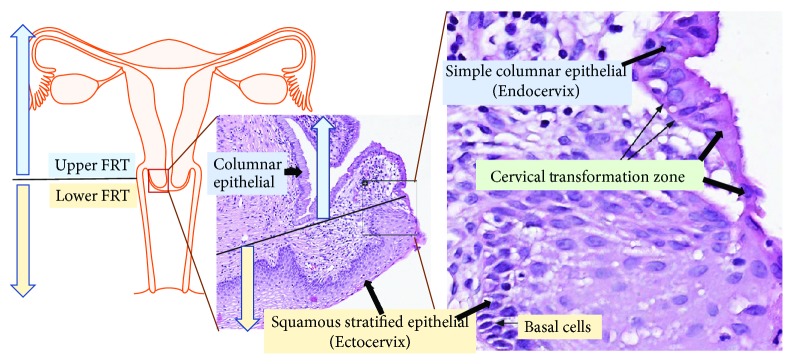
Histology of the female reproductive tract (FRT). This figure illustrates the two immunologically distinct regions of FRT (see left side), i.e., the pathogen-laden lower FRT, shown by the yellow arrow, and the “sterile” upper FRT (see blue arrow) and their respective histological sections (see images in the middle and right side). Image credit of histological section: Human Protein Atlas, available from http://v18.1.proteinatlas.org.

**Figure 2:**
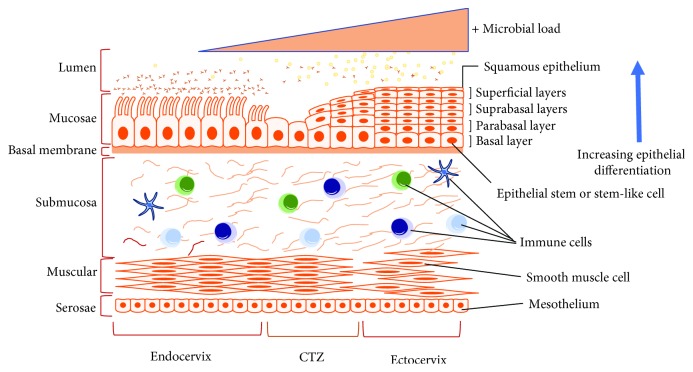
Microscopic illustration of the cervix. The cervix is divided into the two main compartments, namely, the microbe-laden ectocervix and “sterile” endocervix, which are bordered by the cervical transformation zone (CTZ) and their associated immune cells. The ectocervix (and vagina, not shown) has squamous stratified epithelia, while endocervix (and uterus, not shown) has simple columnar epithelia.

The female reproductive tract (FRT) can be divided into two immunological regions, namely, the upper (endocervix, uterus, and oviduct) and lower (vagina and ectocervix) FRT [1]. While the lower FRT is associated with a heavy microbial presence, the upper area is considered “sterile,” although this theory has been recently challenged [2] (Figures 1 and 2). Thus, each of the two regions has unique tissue-specific immunological features and they both undergo specific alterations during different physiological conditions (menstrual, pregnancy, postpartum, and age), and collectively, they create an immune response spectrum with two “opposite” ends [3]. On one end of the spectrum, an immune response essential for protection against infection and other noxious environmental insults is generated. On the other end of the spectrum, and simultaneously, a symbiotic mutualism with commensal microbes and immunological tolerance to allogenic sperm, as well as semiallogeneic fetus, is maintained during pregnancy [3]. These complex immune responses are largely orchestrated and regulated by the sex steroid hormones, and dysregulation of these immune responses can lead to various clinical complications, such as bacterial vaginosis, vaginal candidiasis, cervical cancer, urinary tract infections, and preterm labor [4, 5]. The present review largely focuses on the tissue-specific immunological alterations that take place in the ecto- and endocervix under different physiological conditions.

The human cervix is a barrel-shaped structure with a central canal and measures approximately 2 cm long. The proximal end of the cervix is called internal *os* and opens into the uterus without any obvious features to mark the borders of the two tissues, whereas the distal end is called external os and opens into the vagina [6]. The cervical tissue has two dominant cell types in both endo- and ectocervix consisting of (a) stromal and (b) epithelial cells. These two cell types are separated by a basement membrane [7]. The stromal tissue is predominantly made up of fibroblasts, with smooth muscle, and scattered immune cells, embedded in a collagen-rich extracellular matrix containing hyaluronan and proteoglycans [8]. The present review focuses on the two cell types that play a predominant role in local immune responses, namely, the cervical epithelial and immune cells. Overall, both the cervical epithelial and immune cells from the ecto- and endocervix protect the upper FRT from bacterial infiltration through various mechanisms. (a) The cervical epithelial cell mounts a physical barrier through mucus and epithelial sheets. These cells (epithelial) also generate specific immune responses and collaborate with regular immune cells. (b) The immune cells (resident and nonresident) orchestrate both innate and adaptive immune responses [3, 9, 10].

## 2. The Multi-Immune Facets of Cervical Epithelial Cells

### 2.1. General Overview

Epithelial cells are the “first responders” to come in direct contact with potential pathogens that infiltrate the FRT. As such, they have to be flexible, nimble, and adaptive. Also, an effective communication between epithelial and immune cells is critical in mounting an effective protection against infection. For instance, FRT epithelia, including that of the cervix, are capable of mounting diverse innate and adaptive immune functions. Essentially, the cervical epithelial cells have several immune capabilities that are used to block ascension of pathogens to the upper FRT. Notably, these include (1) mounting a physical immune barrier, (2) secreting antimicrobial, cytokine, and chemokine factors, and (3) exhibiting some of the more specialized innate and adaptive immune functions, such as antigen presentation.

### 2.2. Cervical Epithelial Cell Mounts Physical Barrier against Pathogens

The upper FRT is lined with a single layer of columnar cells, while the ectocervix has a stratified layer of cells covered by squamous epithelial cells. The two types of epithelia (ecto versus endo) are separated by a junction with squamocolumnar cells. The barrier properties of the mucosal epithelia are regulated in part by adhesion molecules which seal off the intercellular space between adjacent cells [11]. Thus, the integrity of epithelial barrier is maintained by intercellular junctions that prevent the invasion of microbes. The intercellular junctions present in the lateral membrane of epithelial cells can be classified into three main types, namely, tight or occluding junctions, adherent junctions (including desmosome and hemi-desmosomes), and gap junctions (communicating junctions). The second physical barrier mounted by the cervical epithelia is the secreted mucus [12].

#### 2.2.1. Cell Junctions in the Cervical Epithelia

**Figure 3:**
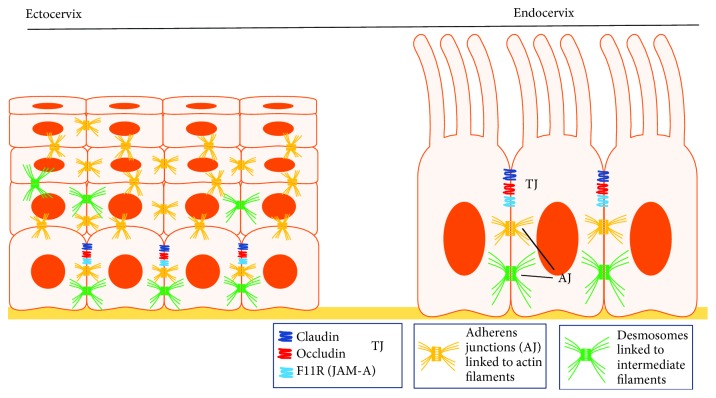
Profile of intercellular junctions in the cervix. This figure shows the profile and intercellular localization of interepithelial adhesion molecules in the ecto- and endocervix. The most “potent” junctions in the stratified squamous epithelium of the ectocervix (and vagina), namely, adherens junctions (AJ), are located in the parabasal layer, just above the basal layer. As epithelial cells transition toward the apical surface, the junctions get “more loose.” In contrast, endocervical epithelia contain tight junctions (TJ) located close to the apical surface and AJ just below the TJ. Desmosomes are the most basal junctions.

An electron microscopy study has demonstrated that classical tight junctions comprise the principal intercellular junctions between the simple columnar epithelial cells in the endocervix [13, 14]. In contrast, the uppermost layers of the stratified squamous ectocervical epithelium are devoid of organized intercellular junctions [13, 14]. Moreover, junctional molecules thought to be key regulators of epithelial permeability, junctional integrity, and leukocyte infiltration, such as F11R (or JAM-A), E-cadherin, occludin, claudin-1, and TJP1 (or ZO-1), are abundant in the human endocervix [13]. A lack of tight junctions in the lower FRT does not only allow movement of small molecules and pathogens between epithelial cells but also enables immune cells, such as epithelial cervicovaginal Langerhans cells or dendritic cells (DC), to do the same. Unsurprising is the fact that the ectocervix and the vagina share a continuous morphologically identical mucosa layer, and therefore, the distribution of junctional molecules is similar in both tissues. According to Blaskewicz et al., the most robust junctions in the stratified squamous epithelium of the ectocervix and vagina lie in the parabasal epithelium, just above the basal layer in contact with the basement membrane [13]. Adherent junctions were particularly abundant within these cells. The integrity of the junctions progressively lessens toward the apical surface, where epithelial cells become cornified, lose all cellular contacts, and are sloughed into the lumen [13]. The structure of tight junctions is affected by hormones [15] as well as cytokines [11]. For instance, our group has demonstrated that exogenous vascular endothelial growth factor (VEGF), a potent angiogenic factor, promotes paracellular permeability in cervical epithelial cells during pregnancy [16]. Besides, tumor necrosis factor-*α* (TNF-*α*), a signaling molecule, decreases transepithelial resistance in uterine epithelial cells [17]. The presence of gap junctions in cervical epithelial cells has been scarcely studied [18, 19] (Figures 1 and 3).

#### 2.2.2. Mucus as the Second Physical Barrier with Antimicrobial Activity

**Figure 4:**
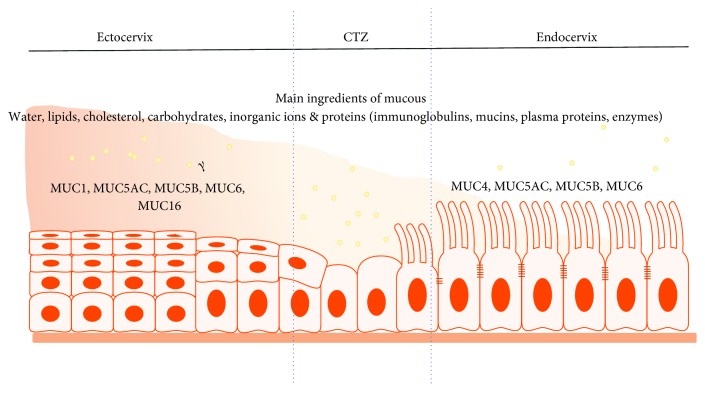
Profile and distribution of cervical mucus. This figure shows the main ingredients of cervical mucus and common and distinct distribution of mucin in the ecto- and endocervix. Mucus provides a physical and chemical barrier against pathogens.

The cervical mucus is produced by goblet cells from pseudoglands and undergoes marked modifications that results in signature biochemical and biophysical characteristics at different stages of the cycle [12, 20]. Mucus is a mixture of various factors whose composition and concentration vary across the cycle and during pregnancy. These factors include water, lipids, cholesterol, carbohydrates, inorganic ions, and proteins (immunoglobulins, plasma proteins, mucins, and enzymes) [12]. The changes in mucus consistency during the menstrual cycle and or pregnancy vary mucus's ability to physically block ascendancy of bacteria while permitting entry of sperm to the upper FRT. For instance, the upper FRT is vulnerable to infection during menstruation and childbirth when the cervix opens to allow passage of menstrual flow and the baby. Therefore, the immune response generated by the cervix has to be flexible and nimble under the varying physiological reproductive conditions, enabling the cervix to play various key roles in female reproduction, notably being the key immune “gate keeper” of the “sterile” upper FRT (Figure 4).

Mucins are large glycoproteins that serve as the gel or glue of the mucus and are, therefore, able to entrap and immobilize pathogens, among other things, depending on the thickness of the mucus [20]. There are more than 20 different mucins and are differentially expressed by goblet cells depending on the location and menstrual status [20]. MUC4 and MUC5 (5AC, 5B) and MUC6 are the major mucin proteins of the endocervical mucus, and their quantity changes during the cycle [21]. Overall, the cervical mucus acts as a semipermeable and antimicrobial barrier, preventing access of pathogens to the upper genital tract while simultaneously permitting sperm entry prior to ovulation [20, 21] (Figure 4).

The antimicrobial and immune constituents of mucus compliment the physical barrier mounted by cervical mucus. These constituents cited earlier include immunoglobulins, complement, antibodies, cytokines, antimicrobial proteins, and immune cells [22], and their concentrations or levels display cyclic fluctuations [22, 23], whereas other molecules, such as IL-6, IL-4, IL-12, IFN-*γ*, and T cell activity, do not have cyclic variation [23, 24]. While these cyclic variation in mucus composition increases vulnerability of the upper FRT as they may result in ascendance of pathogens, the thinning of the mucus during ovulation also allows for easier passage of sperm [12]. The combination of a physical and immune barrier mounted by the mucus and epithelial sheet is perhaps compensatory and potentiate the immune function by for instance slowing particle diffusion while allowing time for biological defense [12].

### 2.3. Cervical Epithelial Cells Secrete Antimicrobial, Cytokine, and Chemokines

Epithelial cells are known to secrete a concoction or cocktail of regulatory factors that either synergize or complement each other, such as cytokines and chemokines and a spectrum of antimicrobials, including lactoferrin, lysozyme, complement, and defensins, along with immunoglobulins A (IgA) and IgG that confer protection against pathogens [25–27]. For instance, the secretion of antimicrobial molecules in the cervical mucus provides an interesting example of the synergy or interdependence between the innate and adaptive immune functions. Several species of bacteria in the mouse uterine lumen bind complement C3 produced by epithelial cells, which (complement C3), in turn, facilitates the binding of IgA and IgG antibodies to the bacteria, ultimately leading to a more efficient phagocytosis of bacteria by neutrophils [28]. Therefore, while the production of complement components is an innate process, the antibodies are part of the adaptive process that leads to protection [9]. It has been shown that the expression of IgA and IgG in uterine tissue is hormonally regulated [27]. The distribution of complement receptor 3 (CR3) on the epithelia of the human female genital tract has been described previously, as well as the production of alternative complement components in primary human ecto- and endocervical cells, which subsequently allows C3b deposition and its rapid conversion to iC3b [29–32].

**Figure 5:**
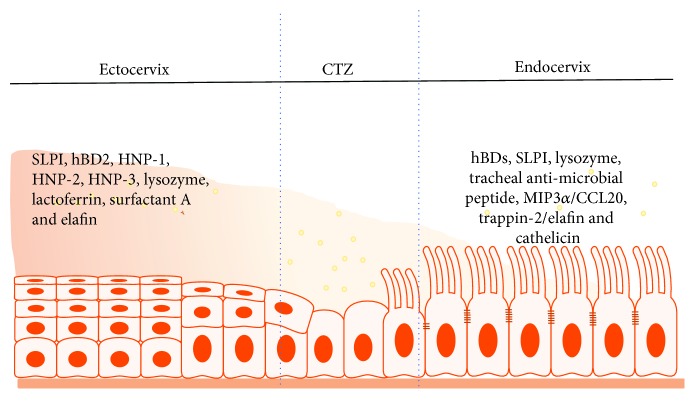
Antimicrobial peptide (AMP) profile and distribution in the ecto- and endocervix. Full names of abbreviations: human *β*-defensins (hBDs), secretory leukocyte protease inhibitor (SLPI), lysozyme, macrophage inflammatory protein-3 (MIP3*α*/CCL20), surfactant A, and elafin (also known as peptidase inhibitor 3).

**Figure 6:**
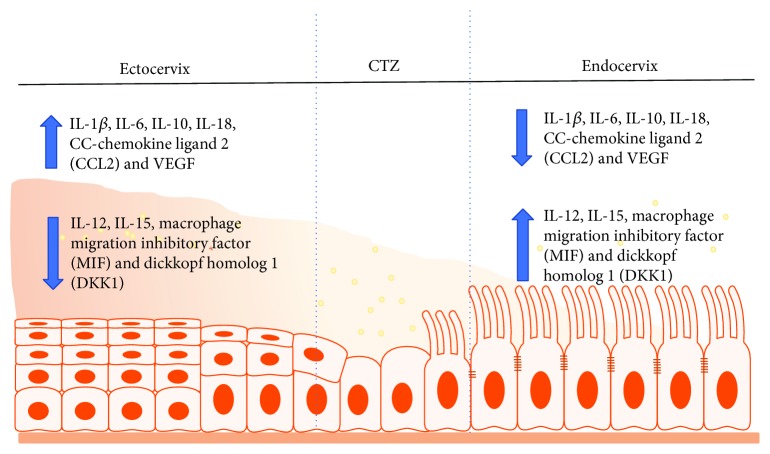
Cytokine profile, distribution, and expression pattern in the ecto- and endocervix. Full names of abbreviations: interleukin (1*β*, 6, 10, 12, 15, 18), vascular endothelial growth factor (VEGF).

**Figure 7:**
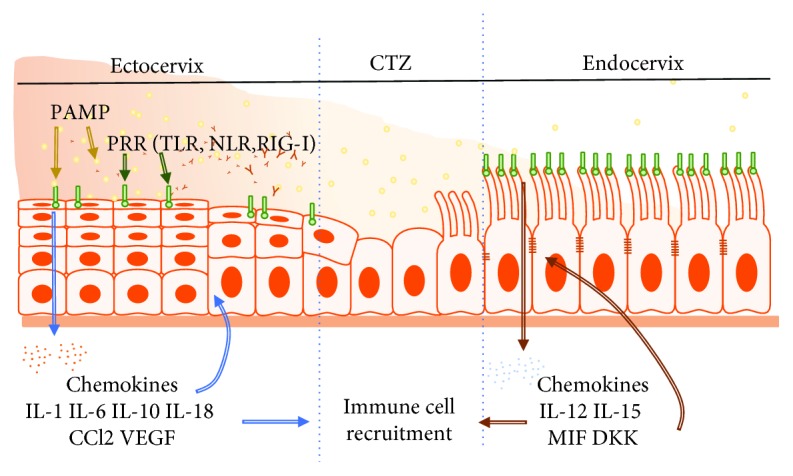
Chemokine profile and distribution in the ecto- and endocervix. Chemokine profile. Full names of abbreviations: interleukin (1, 6, 10, 12, 15, 18), vascular endothelial growth factor (VEGF), macrophage migration inhibitory factor (MIF) and dickkopf homolog 1 (DKK1), pathogen-associated molecular patterns (PAMPs), and pattern recognition receptor (PRR).

Generally, there appears to be a distinction in the composition of the antimicrobial of the lower FRT compared to the upper FRT, a likely reflection of the unique functions of the two tracts [4]. For instance, the upper FRT epithelial cells secrete a battery of antimicrobial peptides, such as human *β*-defensins (hBDs), secretory leukocyte protease inhibitor (SLPI), lysozyme, tracheal antimicrobial peptide, macrophage inflammatory protein-3 (MIP3*α*/CCL20), trappin-2/elafin, and cathelicidin [14], whereas the proteomic profile of the pathogen-laden lower FRT includes SLPI, hBD-2, human neutrophil peptide 1 (HNP1), HNP2, HNP3, lysozyme, lactoferrin, surfactant A, and elafin (also known as peptidase inhibitor 3) [4, 33] (Figure 5). Other common cervical secretions include cytokines, such as IL-1*β*, IL-6, IL-10, IL-18, CC-chemokine ligand 2 (CCL2), and vascular endothelial growth factor (VEGF), whose levels are markedly higher in the lower FRT, while IL-12, IL-15, macrophage migration inhibitory factor (MIF), and dickkopf homolog 1 (DKK1) levels are very low in cervical secretions compared to endometrial secretions [25]. Cytokines modulate diverse physiological, inflammatory, and noninflammatory processes and development, but they also modulate paracellular permeability by restructuring tight junctions through a variety of different signaling pathways. For example, the proinflammatory cytokine tumor necrosis factor alpha (TNF-*α*), transforming growth factor beta (TGF-*β*), and hepatocyte growth factor (HGF) directly impaired tight junctions in a number of epithelial and endothelial cell lines [11], including uterine epithelium [17]. Recently, the current theory on the immune specificity and distinctiveness between the two compartments of the FRT has been challenged [34] since many of the proteins differentially secreted in the upper and lower FRT, such as CCL2, IL-6, IL-10, IL-15, and IL-1*β*, are also involved in immune cell migration and phenotype development [25]. Consequently, the observed differences in the secretion of specific proteins may regulate the variations in immune cell populations across the FRT [4] (Figures 6 and 7).

### 2.4. Cervical Epithelial Cell as an Antigen-Presenting Cell

A typical antigen-presenting cell (APC) has a major histocompatibility complex (MHC) class II molecule located on its surface and exposes a processed antigen to CD4^+^ T cell, which is the basis for driving T cell activation, proliferation, and differentiation into effector cells [35]. APCs are bone marrow-derived and include dendritic cells, macrophages, and monocytes [36]. However, other cell types with demonstrated antigen-presenting capacity include skin Langerhans cells, liver Kupffer cells, and spleen dendritic cells [36]. Antigen presentation by epithelial cells has been demonstrated at several mucosal surfaces, including uterine epithelial cells [37], as well as in uterine stroma and vagina [35]. Within the FRT, epithelial cells express MHC class II molecules [37, 38]. Sex hormones may play a role in regulating antigen presentation [38], which is also regulated by epithelial cell-derived cytokine, as demonstrated by the fact that uterine epithelial cells produce transforming growth factor-*β* (TGF-*β*) that suppresses underlying APCs in the uterine stroma, in response to estradiol (E_2_) [39].

### 2.5. Cervical Epithelial Cells Express Various Pattern Recognition Receptors

We have described earlier how the epithelia of the FRT play a role against pathogens by (a) mounting a physical barrier formed by cell junctions and secreting mucus containing specific proteins, (b) secreting cytokines, chemokines, and antimicrobials, and (c) as antigen-presenting cells. Cervical epithelial cells also recognize pathogen-associated molecular patterns (PAMPs) on microbes (bacteria, parasites, and viruses) through pattern recognition receptors (PRR) such as toll-like receptor (TLR) family, retinoic acid-inducible gene 1 (RIG-I)-like receptors (RLRs), and NOD-like receptors (NLRs) [40–42]. TLR2 and TLR4 detect signals from gram-positive and gram-negative bacteria, respectively [40–42], while NOD1 and NOD2 mediate signals from peptidoglycan moieties derived from gram-positive and gram-negative bacteria, as well as from nonbacterial pathogens, such as viruses and protozoan parasites [43]. RLRs mediate signals from a variety of viruses which leads to the production of IFN-1 and induction of antiviral responses [44]. The level of expression of these receptors is higher in the upper FRT, considered “sterile” compared to the lower FRT, which is pathogen-laden [4, 40]. Specific bacteria-derived ligands (peptidoglycan, lipopolysaccharide) bound to TLR increase the expression of inflammation mediators, such as cytokines and chemokines (mentioned before) [40]. Further, it has been shown that E_2_ alters the profile of cytokine secretion in cultured cervical epithelial cells when TLR2 and TLR4 signaling pathways are activated [40, 42], and the nature of the response varies based on the type of cytokine and duration of the coincubation of cells with E_2_ and TLR ligands [40]. The authors conclude that a complex immune-modulatory role of E_2_ exists at the epithelial surface and that such a role likely enables the mucosal layer of the lower FRT to discriminate between commensals and pathogens and mount appropriate host defense against ascending infection [40]. By contrast, TLR7, TLR8, and TLR9 are evenly expressed throughout the upper and lower FRT. These receptors detect single-stranded RNA (TLR7 and TLR8) and DNA (TLR9). However, the exact signaling mechanisms remain to be elucidated.

### 2.6. The Vaginal Microbiome

The vagina has been compared to a nutrient-rich chamber for microbes [45]. These microbes composed of different species (bacteria, viruses, fungi, and protozoa) play a critical role in maintaining the overall health of the vagina and prevent infections [45–47]. Disruption of this microbial community could lead to vaginal infections, as well as other urogenital complications and adverse pregnancy outcomes, among others [45, 48]. Factors that can change the composition of vaginal microbiota are diverse and include age, hormonal levels, genital infections, and hygiene practices [45, 49]. To date, more than 50 microbial species have been described in the vaginal tract, dominated by Lactobacillus species (70% of the total) [50]. The key members of the lactobacillus species include *L. crispatus*, *L. gasseri*, *L. inners*, and *L. jensenii* [46]. This species (*Lactobacillus*) has received considerable attention because of their protective and probiotic properties [51], which in part is exerted by producing factors that lower pH below 4.5 and inhibit overgrowth of normal facultative anaerobes, namely, lactic acid [52–55]. Other key species found in the vagina include anaerobes (*Gardnerella*, *Atopobium*, *Mobiluncus*, *Prevotella*, *Streptococcus*, *Staphylococcus*, *Ureaplasma*, and *Megasphaera*) and commensal microorganisms, such as the opportunistic fungus, *Candida albicans* [56]. Further studies are needed to understand the underlying mechanism of the interdependence between the FRT microbiota and the host, which will be critical in promoting the reproductive health of women.

## 3. Region-Specific Distribution and Function of Immune Cells in the Mucosae of the Cervix

### 3.1. General Overview

As stated earlier, the vagina and ectocervix, which constitute the lower FRT, are characteristically heavily laden with microbes because they are directly exposed to the external environment, in contrast to the “sterile” upper FRT. As such, it should be expected that these two regions will have different immunological profiles. Further, immune cells in the two regions are overall believed to be primarily regulated by sex hormones during menstrual cycle [4], pregnancy, and postpartum [10] in a site-specific manner, which is dependent on the type of factors present in the local tissue microenvironment, such as growth factors, cytokines, and chemokines [4, 10]. Generally, consistent with mucosae of other tissues of the body, T lymphocytes are much more abundant than B cells in the mucosae of the human cervix [57, 58], and approximately 40% of the cervical T cells are CD4^+^ T cells, the other 60% being CD8^+^ T cells [58]. Within the CD4^+^ T cells located in the cervix, the majority (70%) exhibit an effector memory or effector phenotype, compared to about 60% of the CD8^+^ expressing an effector cell phenotype, which is consistent with the cervix being an effector site for cell-mediated immunity and the tissue residency of cervical CD4^+^ and CD8^+^ T cells [10, 58]. In contrast, natural killer (NK) cells are only 2.7% of the CD45^+^ of total immune cells [3] and CD19^+^ B cells are only 0.9% of the immune cells [3, 58]. However, while Trifonova et al. attest that CD8^+^ T cells are more numerous than CD4^+^ T cells, the specific local distribution of CD4^+^ and CD8^+^ T cells is still debatable [58]. The discrepancies between some studies in the specific proportion of each leukocyte population could, in part, be attributed to the differences in the experimental designs of the studies [57, 58], such as reproductive phase (menstrual cycle vs. menopausal vs. gestation) and sample size [3].

Macrophage, dendritic cells (DCs), and other lymphocyte lineage-negative cells (CD3, CD20, CD56, and CD16) are the major immune cells in the cervix, ranging from 37 to 55% of CD45^+^ mononuclear cells [58]. CD11^+^ macrophages are the dominant cells at approximately 30% of CD14^+^ cells in the cervix. The cervical DCs are slightly more prevalent than peripheral blood DCs, which are less than 1% of CD45^+^ cells [58].

### 3.2. Immune Cells in the Mucosae of the Ectocervix

**Figure 8:**
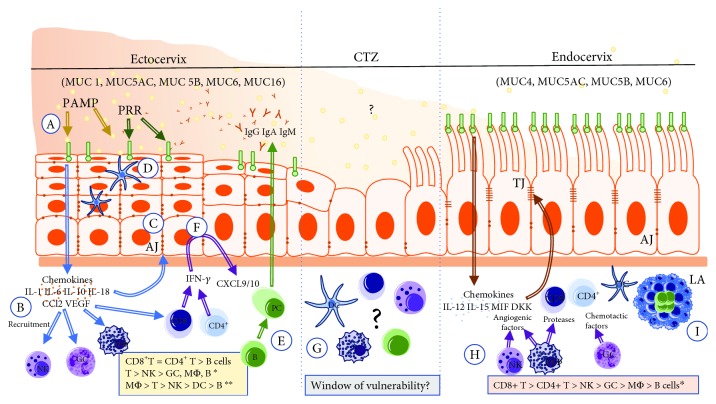
Comprehensive profile of immunity in the three compartments of the cervix. This figure shows the overall innate and adaptive immunity of the pathogen-laden ectocervix and “sterile” CTZ and endocervix, as well as their associated cells and molecules. Full names of abbreviations: interleukin (1, 6, 10, 15, 18), interferon gamma (IFN-*γ*), vascular endothelial growth factor (VEGF), macrophage migration inhibitory factor (MIF) and dickkopf homolog 1 (DKK1), pathogen-associated molecular patterns (PAMPs), pattern recognition receptor (PRR), and cervical transformation zone (CTZ). The illustration on the distribution of leukocytes is a modification from the following manuscripts: Givan et al. [57]; Trifonova et al. [58]; and Zhou et al. [10].

T cells (CD3^+^) are the most abundant leukocytes in the FRT and are concentrated more in the lower FRT than the upper FRT [4]. In the lower FRT, most T cells are believed to have effector memory phenotype [58] and 35% of CD45^+^ mononuclear cells are CD3^+^ T cells. Also, CD4^+^ T and CD1a^+^ immature DCs are significantly more abundant in the ectocervix than in the vagina [58]. On the other hand, the CD4^+^ and CD8^+^ T cells are equally abundant in the ectocervix, in contrast to the endometrium where CD8^+^ T cells predominate [4]. Although NK and B cells are also present in the ectocervix, they are much less prevalent than T cells [4]. NK and granulocytes (CD66b^+^) are more abundant in the upper FRT than in the lower FRT [4]. Both T and B cells are more abundant in the ectocervix than the endocervix, correlating positively with the microbial load [10, 58] (Figure 8).

Because the number of CD8^+^ T, B, and NK cells decrease after menopause, immune cells in the human cervix are likely regulated by hormones [59]. Myeloid APCs are also prevalent in both the human ecto- and endocervix [10]. In the APC compartment, CD14^+^ cells are the most abundant, with the largest populations being CD11c^−^ macrophages followed by CD11c^+^ cells, which encompass tissue DCs and/or monocytes that have not yet been fully differentiated into macrophages [58]. Conventional DC subsets are also detected in the CD14^−^ subset of APCs; however, their abundance, although greater than in the peripheral blood, is much lower than that of the CD14^+^ cells [58]. Despite the characterization of these human cervical immune cells, the underlying regulatory mechanisms of their differentiation and functions by systemic and local immune signals remain to be studied. The fact that Pudney et al. were able to detect higher concentrations of CD8^+^ and TIA1^+^ T lymphocytes in the ectocervix and cervical transformation zone (CTZ) suggests that these could be major effector sites of cytotoxic T lymphocyte responses [60]. Also, because the ectocervix and CTZ contain the highest number of antigen-presenting cells, they could also likely be the best location for the induction of cell-mediated immune responses in the lower FRT [60]. Although B cells are a minor cell population throughout the female reproductive tract, IgG- and IgA-producing plasma cells are predominantly localized in the cervix and, to a lesser extent, the vagina. Specifically, cervicovaginal secretions are characterized by greater amounts of IgG than IgA, and both are hormonally regulated [14].

DCs are the most potent antigen-presenting cells and as such play a major role in the uterine defense [61] by activating naïve T cells, an essential step in initiating adaptive immune response [61]. DCs have been predominantly seen in the basal and suprabasal regions, and their dendritic nature was more prominent in the basal layers of the stratified squamous epithelia of the ectocervix and the CTZ [61]. DCs have also been described in the intermediate layer with no typical dendritic processes [62]. Although the specific function of these cells remain unknown, Rabi et al. have proposed that they maybe nondendritic accessory antigen-presenting cells [62]. The distribution of DCs in the subepithelium is not uniform, and they (DCs) migrate from this location to the epithelium where they encounter the antigens [62].

### 3.3. Immune Cells in the Cervical Transformation Zone (CTZ)

The CTZ represents an abrupt transition between the immunologically distinct regions of the lower and upper FRT, namely, the ecto- and endocervix [60, 63]. It (CTZ) appears to be an immunocompromised region as it is a site of multiple pathogenic conditions, including HIV and metaplastic squamous epithelium, which both develop at the squamocolumnar junction. It is, therefore, rather surprising that few studies have specifically examined cells from this immunologically strategic region. Indeed, it has been shown as a preferential localization of lymphocytes and APCs to the cervical CTZ, in essence, mounting the “last” line of defense and “drawing the line” between the pathogen-laden ectocervix (lower) and the “sterile” endocervix (upper FRT) [60]. Specifically, it has been shown that CD8^+^ and TIA1^+^ T cells accumulate in the CTZ, implying that the CTZ functions as the “last” immunological barrier to ascending microbes. However, and surprisingly, this site, which has a higher concentration of CD4^+^, is the preferred site of HIV-1 infection [60] and greater than 90% of cervical cancers originate from it [64]. It is still unclear why such an immune cell-rich region and strategic immunological “border” or landmark is highly susceptible to diseases and malignant tumor (Figure 8).

### 3.4. Immune Cells in the Mucosae of the Endocervix

The population density of T cells and macrophages in the endocervix is much lower than that of the ectocervix, where CD8^+^ predominates [4, 60]. The endocervix contains numerous IgA^+^ and IgM^+^ plasma cells that mediate humoral immune response [60]. Furthermore, no CD1A^+^ dendritic cells are observed or localized in the epithelia, glands, or lamina propria of the endocervix [60]. Also, interestingly, no differences were observed in the number and pattern of leucocyte localization during the proliferative versus secretory phases of the menstrual cycle or in postmenopausal women [60]. Nonetheless, sex hormones are believed to influence the functions of immune cells [60] (Figure 8).

As stated earlier, the ecto- (or lower FRT) and endocervix (or upper FRT) are two immunologically distinct regions, with some variation in the profile of immune cell subpopulations. For instance, CD3^+^ and B cells are twice as much in the ectocervix compared to the endocervix [58], and additionally, both CD4^+^ and CD8^+^ T cells are more prevalent in the ectocervix than the endocervix [58]. However, there are also some similarities between the two regions. For instance, the number of NK cell subsets of CD56^bright^CD16^−^ and CD56^dim^CD16^+^ is not significantly different between the endo- and ectocervix, and generally, the IL-22-producing NK cells, known to play a key role in mucosal immunity, are very rarely detected in the cervix [58]. A summary of the review is provided in Figure 8.

## 4. Immune Profile in the Ecto- and Endocervix during Different Reproductive Phases

### 4.1. General Overview

Estradiol (E_2_) and progesterone (P), which are secreted by the ovaries during menstrual cycle and pregnancy, are the primary regulators of the cellular function (epithelial, fibroblasts, myometrial, and immune) of the FRT [57, 65]. The immune cells, which include natural killer (NK) cells, macrophages, dendritic cells, and T cells, are the main cells responsible for fighting infections, while simultaneously ensuring a successful conception and subsequent development and birth of the fetus [4, 40, 66]. Specifically, the immunological effects of the two hormones (E_2_ and P) are contradictory, i.e., while P exerts anti-inflammatory effects by inducing the transcription of proteins and growth factors that mitigate inflammation and promote repair in mucosae [66], E_2_, on the other hand, regulates the activity of different inflammatory transcriptional factors, such as AP-1, SP1, STAT, and vitamin D receptor in human uterine cells [44], as well as alters the immune response of cervical epithelial cells [40].

### 4.2. Menstrual Cycle

The menstrual cycle is divided into two main phases: (1) the proliferative (FRT) or follicular phase (ovarian) and (2) the secretory (FRT) or luteal phase (ovarian) [67]. As stated earlier, the changes and fluctuations in hormonal levels during the menstrual cycle have a profound region-specific effect on the functional status of the immune system of the FRT, including the cervix [4].

For instance, at midcycle (ovulation), when E_2_ levels are high, the production of antimicrobial peptides is inhibited in the lower FRT, thus creating a window of vulnerability for infection [68]. On the other hand, the activity of cytotoxic cells in the upper FRT is reduced at a time when the production of antimicrobial peptides is enhanced, thus optimizing conditions for successful implantation [58, 68]. Furthermore, during the proliferative stage of the menstrual cycle (prior to ovulation), cervical/vaginal secretions contain higher levels of antimicrobial peptides compared to other phases of the cycle [68], thus complementing and reinforcing its defense barrier capabilities against microorganisms, besides its immunomodulatory effects.

The proliferative phase is also characterized by angiogenesis and the regeneration of the endometrial and cervical tissue and the concentration of immune cells in the endometrium diminish during this time [65]. In contrast, in the lower FRT, fluctuations in the levels of sex hormones do not affect the number of immune cells, which remain constant throughout the cycle [60]. Around the midcycle, when uterine angiogenesis peaks, recruitment of innate immune cells such as NK cells, neutrophils, and macrophages in the uterus is facilitated [4]. This recruitment is mediated by cytokines, chemokines, and growth factors, which are mainly produced by uterine endometrial cells and fibroblasts under the influence of both sex steroids [65]. Overall, 40-50% of leukocytes in the FRT are T cells [57, 58, 69], and during the proliferative phase, most T cells in the endometrium are dispersed or are concentrated in small aggregates in the stroma or as epithelial lymphocytes [4]. Lymphoid aggregates (LA) structurally have a core of B cells in the center surrounded by CD8^+^ T cells and encapsulated by macrophages [70]. These immunoaggregates are formed in the endometrium but not in the ectocervix or the lower FRT, in the absence of an infection [70]. They (LAs), however, peak in size midcycle and persist during the secretory phase and may provide protection against secondary infections or may play a role in maintaining the T cell repertoire and prevent the loss of resident memory cells during menstruation [4, 70].

During the late secretory phase and menstruation, which is associated with a decrease in sex steroids and subsequent initiation of inflammatory response, the number of immune cells increases in the endometrium [57, 65, 71]. These inflammatory responses include decreased prostaglandin metabolism, loss of protection from reactive oxygen species (ROS), and increased synthesis of proinflammatory prostaglandins, cytokines, chemokines, and matrix metalloproteinases (MMP), which result in recruitment of leukocytes. Collectively, these actions lead to tissue breakdown and the characteristic bleeding associated with menstruation [71].

*Macrophages* on the other hand only represent about 10-20% of the FRT leukocytes [4, 57, 58]. However, they are more abundant in the stroma of the endometrium than the endocervix or the ectocervix, and their number remains stable during the proliferative phase [4]. *Uterine NK* cells represent around 30% of leukocytes during the window of implantation and increase in number during the secretory phase and populate the endometrium before pregnancy [65, 72]. It is still unclear how exactly the number of NK cells increases [4]. Overall, a plethora of immune cells, including neutrophils, NK cells, macrophages, eosinophils, and DCs, migrate into the uterus in response to hormonal changes [71, 73]. For instance, P withdrawal triggers menstruation and an inflammatory response in the endometrium, leading to an influx of leukocytes that mediate tissue breakdown and repair [4].

### 4.3. Postmenstrual Cycle

During menopause, the fluctuation of sex hormones declines and their levels remain low and constant [74]. These hormonal alterations lead to various changes, notably a progressive dysregulation of the systemic immune system that leads to increased susceptibility to infections and malignancies and reduced responses to vaccination and increased general inflammation [75]. Moreover, women appear to lose their immunological advantage after menopause, since in aging, they are more susceptible to infections and to develop inflammatory diseases [68]. The specific changes in the systemic immune system include a decreased CD4^+^ : CD8^+^ cell ratio, increased number of differentiated effector and memory T cells, depletion of naive T cells, and decreased frequency of B cells [75]. Natural killer (NK) cells, which fight against viral pathogens and tumors, increase with age [75]. However, they (NK cells) exhibit a decrease in cytotoxicity and in their ability to produce cytokines [75, 76]. Aging is also associated with increase in levels of proinflammatory cytokines, which consequently leads to an increased inflammatory state [75]. Specifically, in postmenopausal women, there are higher levels of serum proinflammatory cytokines, such as monocyte chemoattractant protein 1 (MCP1/CCL2), tumor necrosis factor alpha (TNF*α*), and IL-6 and free radicals [59]. On the other hand, CD4^+^ T and B lymphocytes and cytotoxic activity of NK cells typically decrease [59] (Table 1).

A plethora of immune factors, dependent on estrogen, are downregulated during menopause. These include loss of toll-like receptor (TLR) function, decreased secretory antimicrobial components, lack of cervical mucus production, alterations in commensal lactobacilli and acidity of vaginal microenvironment [75], and a general senescence of immune cells [77]. Specific changes in the cervix associated with senescence of immune cells include loss of dendritic cells and resident memory T cells [77]. Further investigation is needed to evaluate the immune response of postmenopausal woman in the cervix.

## 5. Conclusions

The cervix plays a complex balancing act in female reproduction under the influence of sex steroid hormones, by acting as the pathogen and germ/fetal “gatekeeper,” i.e., on the one hand it blocks ascension of pathogens to the upper FRT, while allowing that of the sperm and elimination of menstrual fluid and fetal passage at birth. Dysfunction of this complex role could lead to serious gynecological and obstetrical complications, such as development of polyps and cysts, dysplasia, cancer and cervical incompetence, urinary tract infections, bacterial vaginosis, candidiasis, and viral infections. Development of effective therapies to these disorders will depend on a comprehensive understanding of the underlying molecular mechanisms governing the immune response of local mucosal and immune cells during the different female reproductive phases. New technologies, such as metagenomics, metabolomics, and transcriptomics, should help delineate these complexities.

## Figures and Tables

**Table 1:** Immune profile of the cervix during menopause.

↓ levels of sex steroid hormones lead to
A. ↑ general inflammation
(i) ↑ levels of proinflammatory cytokines MCP1, TNF*α*, and IL-6
(ii) ↓TLR
B. ↓ decreased immune response
(i) ↑ susceptibility to infections and malignancies
(ii) ↓ response to vaccination
(iii) ↓ decreased CD4 : CD8 ratio
(iv) ↑ number of differentiated memory cells and effector T cells
(v) ↓ frequency of B cells
(vi) ↑ NK but with low cytotoxicity and ability to produce cytokines
(vii) ↓ antimicrobial peptides
(viii) ↓ cervical mucus
(ix) ↓ senescence of immune cells
(x) ↑ loss of DC in the cervix
(xi) ↑ loss of resident memory T cells
